# Lucid Dreaming Brain Network Based on Tholey’s 7 Klartraum Criteria

**DOI:** 10.3389/fpsyg.2020.01885

**Published:** 2020-07-29

**Authors:** Brigitte Holzinger, Lucille Mayer

**Affiliations:** ^1^Institute for Consciousness and Dream Research, Vienna, Austria; ^2^Certificate Program Sleep Coaching, Medical University of Vienna, Vienna, Austria

**Keywords:** Klartraum, lucid dreaming, pre-lucid, consciousness, free will, self-awareness, choice, brain regions

## Abstract

Lucid dreaming refers to a dream state characterized by the dreamers’ awareness of being in a dream and being able to volitionally control its content. The aim of this study was to describe and model neurophysiological evidence for the seven awareness criteria of lucid dreaming based on those proposed by Paul Tholey. Each of the awareness criteria was analyzed separately with regard to its underlying neurocircuits. We hypothesized that not one, but several regions are involved in the state of lucid dreaming. Our results have shown a satisfactory overlap of the awareness criteria and the brain regions activated. During lucid dreaming, a brain network seems to emerge, that is something other than the sum of its parts. Further research is needed to understand the psychoneurological underpinnings of lucid dreams.

## Introduction

Lucid dreaming (LD) is a fascinating research topic and has attracted many enthusiasts. Unfortunately, the scientific field is still lacking a comprehensive definition of LD.

The term “lucid dream” was coined by the Dutch psychiatrist Frederik Willems van Eeden (Holzinger et al., 2006) who reported that in lucid dreams, “the reintegration of the psychic functions is so complete that the sleeper reaches a state of perfect awareness and is able to direct his attention, and to attempt different acts of free volition” (Van Eeden, 1913). The phenomenon of LD is generally understood as the fact that a dreamer is aware that he/she is dreaming while dreaming (LaBerge, 1980; Spoormaker and van den Bout, 2006). Tholey and Utecht (1987) defined additional criteria explaining LD, such as awareness of freedom of decision, memory of the waking state, and full intellectual abilities. Gackenbach and LaBerge (1988) expanded the original definition by requiring the dream to be ongoing, because sometimes the dreamer wakes up upon realising his/her state, and that would be defined as a pre-lucid dream (PLD) instead. Deirdre Barrett (1992) in which the following four criteria were examined: (1) the dreamer is aware that he/she is dreaming, (2) objects disappear after waking, (3) physical laws need not apply in the dream, (4) the dreamer has a clear memory of the waking world.

For now, we preserve the definition according to Tholey (1977) and LaBerge et al. (1981). Lucid dreaming is a dream state characterized by the dreamer’s awareness of being in a dream and the awareness of choice (LaBerge, 1980a, b, 1985; LaBerge and Rheingold, 1991; Holzinger et al., 2006). Tholey (1980, 1981) however, being a German Gestalt Theorist, called the lucid dream “Klartraum,” or “Dream of Clarity” as Holzinger refers to it (Holzinger, 2009). Upon self-exploration of his dreamlife he described seven criteria of a “Klartraum” to be distinguished from a “Non-Klartraum” (Tholey, 1980, 1981). He declared criteria 1–4 as essential for a “Klartraum,” while criteria 5–7 are optional and do not make a “Klartraum” by themselves.

1. Clarity that one is dreaming.
2. Clarity about the freedom of choice (for experiments on the topic see: Libet et al., 1983; Fried et al., 1991; Haggard and Eimer, 1999; Soon et al., 2008; Liljenström, 2015; Liljenström and Nazir, 2016; for an overview see: Baumeister et al., 2010; Caruso, 2012).
3. Clarity of consciousness.
4. Clarity about the waking life.
5. Clarity of perception.
6. Clarity about the meaning of the dream.
7. Clarity recollecting the dream.

The seven criteria used in this article are based on Tholey’s, however, we used an adapted version (Holzinger, 2014) that fits the Gestalt theory terminology better (Yontef, 1993). We suggest these criteria are more closely related to newer neurophysiological findings and reportings of lucid dreaming experiences. Awareness being a lasting state seems to describe the process of a lucid dream better, compared to a moment of clarity which tends to be momentary. Nevertheless, the following criteria are in its core the same as those proposed by Tholey.

1. Awareness of (spatial) orientation.
2. Awareness of the capacity of choice.
3. Awareness of (intense) concentration – (awareness of “flow” Csikszentmihalyi et al., 2014).
4. Awareness of identity (the “I”).
5. Awareness of the dreaming environment.
6. Awareness of the meaning of the dream.
7. Awareness of memory.

Criteria 1 refers to the self-reflective capacity to appreciate the dream as a dream, by recognizing the dream environment and localizing oneself in it. As Tholey stated, the consciousness of being in a dream (or in our case orientation) is not sufficient for a dream to become lucid. The capacity of choice is what changes a dream (Tholey, 1980). Therefore, we suggest that awareness criteria 1 and 2 are crucial for the experience of LD. If only 1 awareness criteria applies, we should be speaking of a PLD (Green, 1968) since all imply some level of self-reflective capacity which in turn can lead to further cognitive capacities. Awareness criteria 5–7 are not essential for the definition for the PLD, LD and “Klartraum,” but can be part of a PLD (distinguishing the PLD from the non-lucid dream), the LD and the “Klartraum” (Holzinger, 2004), describing this extraordinary state and its potential. The definition of LD is still a work in progress and we hope that the discussion about the definition of a pre-lucid, a lucid dream and Klartraum will gain momentum in the scientific community.

Additionally, we would like to propose the value of the seven awareness criteria of LD/ “Klartraum,” tracing back to Tholey (1977, 1980, 1981) in another field of research regarding lucid dreaming, namely the search for correlations of the LD state with specific cortex activation patterns of the brain. Our proposition here is that the “lucid” experience requires changes not in one but several areas of the cortex, and consequently the emergence of a brain network. Lewes (1875) defines emergence as follows: “The emergent is unlike its components insofar as these are incommensurable, and it cannot be reduced to their sum or their difference” (p. 413). It therefore occurs when an entity is observed to have properties its parts do not have on their own and in this case, the brain network is the new entity. Therefore, we assume a model of brain activation on the basis of the seven awareness criteria first described by Paul Tholey, and call it the “lucid brain model,” trying to integrate the varying results of research projects within the last decades.

First, former findings regarding the general difference in brain activity during REM sleep and LD will be discussed, the matter of consciousness in LD will be introduced, and finally neuroscientific evidence for each of our seven proposed awareness criteria will be presented.

## A Brain Network in Lucid Dreaming

### From a Non-lucid to Lucid Dreaming Network

There has been a great deal of speculation about the nature of changes during sleep in the known networks identified by fMRI resting state functional connectivity studies (for an overview see Raichle et al., 2001; for reviews see Fox et al., 2013; Picchioni et al., 2013; Pace-Schott and Picchioni, 2017; Baird et al., 2019). Although the review by Baird et al. (2019) is the only one dealing directly with lucid dreaming, other studies, particularly those examining REM (Fox et al., 2013) have relevance to network-based theories on what is happening during lucidity.

During REM sleep, neural activity in the *brain stem*, *thalamus*, *amygdala*, and *extrastriate temporo-occipital cortices* increases, while other structures such as the *dorsolateral prefrontal cortex* and the *precuneus* show deactivation (Dresler et al., 2012). Hobson and Pace-Schott (2002) have theorized that this activity pattern might reflect visual hallucinations, emotional intensifications, and cognitive abnormalities typically experienced in dreams (Dresler et al., 2012). Deeper areas of the brain (limbic system, memory structures, arousal system) continue to play a role during the lucid dream state but will not be discussed in this article. We focus on those areas reactivated during LD in contrast to non-lucid REM sleep, especially frontal brain regions (Hobson and Pace-Schott, 2002). This recovery of reflective cognitive capabilities is likely to be the hallmark of LD (Dresler et al., 2012). Lucid dreamers report being in possession of all their cognitive faculties (Carskadon, 1995) and recent quantitative EEG data findings support the theory that the “wake-like intellectual clarity is paralleled by neural activations in frontal and frontolateral regions” (Dresler et al., 2012). Voss et al. (2018) found that lucidity was accompanied by an increased activation of the *frontal lobes* compared to regular REM-sleep dreams, regarding both synchronicity and consciousness-related frequencies (40 Hz). PET data also shows cognitive control in dreams to be associated with an activation of certain *frontal cortex* components (Shapiro et al., 1995). However, this does not imply that non-lucid dreams completely lack activation in *frontal regions*. Siclari et al. (2017) found that high-frequency frontal EEG activity (20–50 Hz) is higher in dreams that involve “thinking” rather than “perceiving” – which should be more often the case in LDs compared to non-lucid dreams, while parietal activation is higher in “perceiving” dreams. *Frontal lobe* functions include various tasks such as future planning, self-management and decision making, the integration of information from various sources, processing thoughts into words, voluntary movement, categorizing and making sense, forming memories, manage attention, impulse control, personality and empathy. Koch et al. (2016) on the other hand suggest that while frontal brain regions might be involved in directing attention or monitoring and co-vary with consciousness, the conscious experience itself relies on a temporo-parietal-occipital cortical “hot zone.” Therefore, increased activation of the frontal brain regions and temporo-parietal-occipital regions during LD compared to non-LD seem to have numerous effects on conscious awareness, influencing all seven components.

### Conscious Awareness During Lucid Dreaming

At this point, we would also like to emphasize the notion of consciousness in sleep regarding the understanding and the consequent definition of LD as Harry Hunt did in 1995 (Hunt, 1995) and Jennifer Windt in 2011 (Windt and Noreika, 2011).

Consciousness during regular dreams is thought to be mostly primary, or “characterized by a primitive, animistic style of thinking” (Carhart-Harris and Friston, 2010; Hobson and Voss, 2010). William James claimed that reflective awareness is an immanent part of the waking state while dreaming on the other hand lacks this capacity (James, 1981) and other influential dream researchers supported this theory (Freud, 1960; Hobson, 1988). However, newer findings suggest that rational thinking can be part of non-lucid dreaming as well (Cavallero and Foulkes, 1993) and dreams may be accompanied by a varying degree of insight and subjective control (Voss et al., 2018). Dresler et al. (2014) found that experienced volition was significantly higher during waking state and LD compared to non-lucid dreaming, and that the expression of different aspects of consciousness varies across states: while planning ability was most pronounced during wakefulness, intention enactment was most pronounced during LD, and self-determination most pronounced during both wakefulness and LD. Currently, there is no consensus whether dreaming cognition differs greatly from waking cognition, however, even during a mind wandering waking state, executive *prefrontal cortex* (PFC) regions are significantly more activated than during REM-sleep dreams (Fox et al., 2013).

We do suspect different stages of consciousness and a lucid dreamer does show higher cognitive abilities and reflective awareness than a non-lucid dreamer overall. Empirical data supports the assumption that LD may be defined as a hybrid state, which is still partially ruled by lower level consciousness (Voss et al., 2009; Dresler et al., 2012; Voss et al., 2018). This might be the reason that lucid dreams are “happening” as a result of the subconscious, instead of being “created” in the first place. Like all dreams, they are a reflection of ourselves and our lives. Both lucid and non-lucid dreams may involve a “thinking” dimension as well as a “perceiving” or “experiencing” dimension.

Two brain networks have been proposed in the study of consciousness, which seem to anti-correlate and cause a shift between externally and internally directed awareness (Fox and Raichle, 2007): the *Default Mode Network* (DMN; Raichle et al., 2001) and the *Dorsal Attention Network* (DAN; Corbetta et al., 2000). When the attention system is more active the organism’s attention is shifted to external stimuli, and conversely, when the DMN is more active the attention shifts inwards, e.g., to mental imagery (memory reprocessing or future imagination). Paradoxically, the inward shift of attention does not imply an increase in interoceptive sensations (e.g., taste, smell, digestion, pain) but only a shift to imagined visual and auditory content relative to actual empirical content (Pace-Schott et al., 2019). Recently, a third network has been introduced which could explain the emergence of lucidity, the *Frontoparietal Control System*, which seems to integrate information from DMN and DAN (Vincent et al., 2008). The DMN includes the *precuneus*, the *medial prefrontal cortex* (mPFC), and the *left and right inferior parietal cortices* (Raichle et al., 2001) while the DAN is comprised of the intraparietal sulci and frontal eye fields. The LD state seems to arise when DMN and executive functions are active at the same time. The *executive control network* (ECN) including *dorsolateral PFC*, *intra-parietal sulcus*, the salience network (*anterior insula* and *orbitofrontal cortex*), and the cingulo-opercular network (including *anterior cingulate* and *frontal operculum*) is a structure responsible for executive functions and might play a role in LD (Dosenbach et al., 2006).

#### Awareness of (Spatial) Orientation

High frequency activity in the *right posterior parietal cortex*, a region active during spatial perception and visuospatial attention, was associated with the report of a spatial setting in dreams (Siclari et al., 2017). Dream experience in which the dreamer reports a sense of movement were shown to be associated with an increase in high-frequency activity in the area of the *right superior temporal sulcus* (Siclari et al., 2017). This region is involved in the perception of motion and in viewing body movements. Dresler et al. (2012) found activation in the *bilateral cuneus* and *occipitotemporal cortices* during LD. These areas are part of the ventral stream of visual processing, which is involved in several aspects of conscious awareness in visual perception (Rees et al., 2002). According to Dresler et al. (2012) these findings support an exceptional brightness and visual clarity of the dream scenery which have been reported by lucid dreamers. Furthermore, Holzinger et al. (2006) found increased *parietal beta activity* during LD. One specific part, the *temporo-parietal area*, integrates visual, tactile, proprioceptive and vestibular information, and therefore contributes to self-consciousness and own-body imagery (Blanke and Mohr, 2005). If this region is disrupted during waking with magnetic or electrical stimulation, out-of-body experiences can be induced, which are defined as a subjective sensation of being outside one’s own body and may occur with or without viewing the own body (Blackmore, 1982; Blanke and Mohr, 2005). These results, together with the higher activation of meta-cognitive brain areas, possibly supply evidence for the awareness of spatial orientation, the awareness of the dream environment, and the option to navigate in it. This includes the awareness of being in a dream – which is Tholey’s first criteria but is also inherent to our first awareness criteria.

#### Awareness of the Capacity of Choice/Deciding/Expectation/of Being in Charge

Lucid dreamers are often able to act voluntarily within the dream upon reflection or in accordance with plans decided upon before sleep (Carskadon, 1995). However, Stumbrys et al. (2014) have shown that lucid dreamers are only able to remember their intentions half of the time, with half of those remembered intentions being successfully executed. The *right dorsolateral PFC* has been associated with self-focused metacognitive evaluation (Schmitz et al., 2004). Metacognition in this case refers to the “awareness of the awareness,” or higher order consciousness, which is present in LD (Sinclair, 1922; Voss et al., 2018). This might explain the capability of making choices. Furthermore, meta-cognitive evaluation might be the reason for being aware of one’s identity and metacognition includes metamemory, the awareness of one’s memory. The increased activation of the *right dorsolateral PFC* during LD compared to non-LD could be essential for lucidity and has been documented in empirical studies (Nofzinger et al., 1997; Voss et al., 2009; Dresler et al., 2012). Dresler et al. (2012) further observed that *bilateral frontopolar areas* are activated during LD. The *frontopolar cortex* (FPC) has been related to the processing of internal states, e.g., the evaluation of one’s own thoughts and feelings (Christoff et al., 2003; McCaig et al., 2011). While emotionality in normal REM sleep dreams usually resembles “unconscious affect,” referring to “valenced good/bad reactions that occur in the absence of conscious awareness” (Winkielman and Berridge, 2004) the *ventrolateral PFC* is reactivated during lucid dreams and seems to increase self-conscious emotions and a down-regulation of unconscious affect (Clore and Ketelaar, 1997) resulting in reduced negative (and perhaps overall) emotionality compared to normal dreams (Voss et al., 2018). These findings might explain why lucid dreamers are willing to change dream content. Since they become aware of the negative feelings a dream provokes, they try to change it into something more cheerful. FPC activity has also been correlated with a diverse range of other cognitive processes, including multitasking, implementing task sets, future thinking and prospective memory, exploratory decision making, deferring goals and cognitive “branching,” episodic memory retrieval and detailed recollection, evaluating counterfactual choice and facing uncertainty or conflict, complex relational and abstract reasoning, integrating outcomes of multiple cognitive operations, coordinating internal and external influences on cognition, evaluating self-generated information (Boschin et al., 2015). The possible activation of all these cognitive processes during LD might explain the awareness of the option to make sound choices based on thoughts, emotions and memories and individual preferences.

#### Awareness of (Intense) Concentration – A State of “Flow”

Lucid dreaming is characterized by a reflection on one’s own state of mind and not driven by the attention to the external dream scenery, which might lead to a state of more intense concentration or even “flow experience.” Like in an awake flow state, the dreamer is completely absorbed in their current activity, and has a sense of personal control or agency over the situation or activity, as compared to a state of confusion or semiconsciousness (Tholey, 1981). Additionally, Voss et al. (2018) found that LD differs from non-lucid dreams regarding the positivity of emotions, which might be relevant since the “flow” state is experienced as a very positive one. The flow experience as well as LD are accompanied by hormonal reactions, including norepinephrine, acetylcholine, dopamine, and serotonine (Yuschak, 2006). Acetylcholine has been shown to enhance cognitive function and learning ability and can also enhance LD (Bazzari, 2018; LaBerge et al., 2018). It seems to do so by allowing you to move directly from the waking state into a vivid dream state without losing consciousness (Yuschak, 2006). Dopamine plays an important role in dream recall for REM-dreams (De Gennaro et al., 2016) and might increase the control that a dreamer has within a lucid dream by substantially increasing confidence and motivation levels (Mohebi et al., 2019; Yuschak, 2006). Together with norepinephrine it boosts focus, increases the ability to connect and integrate information, facilitates pattern recognition and problem solving – in case of LD, it might also enhance the ability to recall details and memories from waking life while within the dream (Yuschak, 2006). This allows maintaining constant attention on accomplishing any goals, experiments, or other assignments that you have prepared for the dream. Yoshida et al. (2014) found that during a flow state, the concentration of oxygenated hemoglobin (oxy-Hb) was significantly increased in the *right* and *left ventrolateral PFC*. They also found a significant increase in oxy-Hb concentration in the *right* and *left dorsolateral PFC*, *right and left frontopolar areas*, and *left ventrolateral PFC* while participants were filling out the flow state scale after performing a task in the flow condition. These areas have been found to show increased activation during LD, which supports the LD-flow hypothesis. In conclusion, flow is associated with activity of the PFC, and may therefore be associated with functions such as cognition, emotion, maintenance of internal goals, and reward processing. Therefore, the flow experience shares many characteristics with the LD state.

#### Awareness of Identity – The “I” Without Which There Would Be No Dialogue

Studies have found that lucidity is related to a change on the degree of self-related processing and the type of self-presentation (Metzinger, 2004; Windt and Metzinger, 2007). Self-awareness is thought to be supported by the DMN, its activation leads to an inward shift of attention and has been found to be a hallmark of the REM dreaming state. Accordingly, Dresler et al. (2012) found that the strongest increase in activation during lucid compared to non-lucid REM sleep happened in the *precuneus*. This brain region is also a part of self-referential processing, such as first-person perspective and experience of agency (Cavanna and Trimble, 2006). Holzinger et al. (1998) found that the *left parietal lobe* was also more activated during LD, that area of the brain being related to semantic understanding and self-awareness. The *insula* is another relevant brain structure that lays between frontal, parietal and temporal cortex. Its functions are still investigated, but seem to include control of conscious awareness, motor control, perception and self-awareness (Craig and Craig, 2009). We suggest that this area of the brain might also play a role in LD, however, this is only speculative and requires further exploring. The awareness of the “I” is of course closely related to the awareness of memory, explained in section “Awareness of Memory,” which determines to a great part what the dreamer might decide, wish for or act upon when able to take control of the dream.

#### Awareness of the Dreaming Environment

The awareness and memory of a spatial dreaming environment can be part of non-lucid dreams as well, and is associated with high frequency activity in the *right posterior parietal cortex* (Siclari et al., 2017). However, while regular REM-sleep dreams usually involve an activation of the DMN and not the DAN, during LD, a higher connectivity between those networks evolves and the *Frontoparietal Control System* starts to integrate information from both. Awareness of the environment may be supported by this collaboration of DAN and ECN and the connectivity between frontal and parietal nodes in DAN, DMN, and ECN seems to reflect consciousness that is required for information integration (Picchioni et al., 2013). Together with those findings discussed in section “Awareness of (Spatial) Orientation,” the awareness of the dreaming environment during LD might be explained.

#### Awareness of the Meaning of the Dream

General frontal activation might be the reason for the ability to add meaning to a dream by integrating memory, identity and the dreaming environment into a whole. Based on empirical and theoretical findings, we suggest that a dream becomes meaningful by an integration of emotional content (limbic system), memory (hippothalamus and related structures) and brain structures involved in identity (see section “Awareness of Identity—the “I” Without Which There Would Be No Dialogue”). This might be possible due to an activation of the DMN and executive functions returning when accessing the state of LD compared to non-LD.

Furthermore, meaning is typically added to something by using words, categories and logical thought. Several areas of the *parietal lobe*, which is more active during LD, are important in language processing. The left *parietal-temporal* areas have been found to be relevant for verbal memory and the ability to recall strings of digits (Warrington and Weiskrantz, 1978). *Insula* activity increases in case of unclear images and perceptive input (Lamichhane et al., 2016). We suggest that the *insula* might enable the lucid dreamer to make sense of the dream images. Furthermore, the *insular cortex* plays a role in developing a sense of the physiological condition of the entire body (introception) by collecting internal cues such as the beating of the heart, and related signals provide a basis for time perception (Craig, 2009). Üstün et al. (2017) found activity in the *right dorsolateral prefrontal* and *right intraparietal* cortical networks, together with *the anterior cingulate cortex* (ACC), *anterior insula* and *basal ganglia* during time perception. Meta-cognitive abilities, language processing, as well as time perception might play a role when adding meaning to a dream.

#### Awareness of Memory

Lucid dreamers are often able to remember previous LD experiences as well as the conditions of their waking life (Holzinger et al., 2015). Dresler et al. (2012) found the *dorsolateral prefrontal cortex* and *parietal lobules* to be active during LD, which may reflect working memory demands (Smith and Jonides, 1998). In normal dreams, on the contrary, working memory is strongly impaired (Hobson and Pace-Schott, 2002). The activation of the working memory could allow lucid dreamers to analyze the dream content in relation to their identity, memory and dream environment and decide and plan behaviors according to individual preferences. Ogilvie et al. (1978) found a global increase in the percentage of alpha band (8–12 Hz). This supports the hypothesis that LD is an intermediate stage between REM-sleep and waking. Alpha waves are typical for a state of relaxation and focus and are ideal for learning and memory retention (Makada et al., 2016). In this case, however, follow-up EEG studies found no significant differences in alpha power (LaBerge, 1988) or that only PLDs differed in alpha-power (Tyson et al., 1984).

## Discussion

**FIGURE 1:**
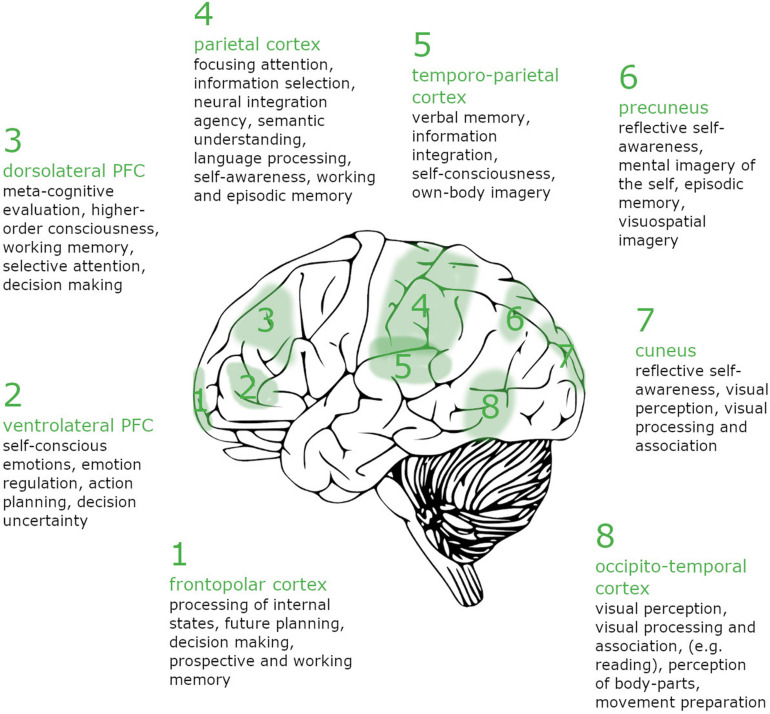
Brain regions showing increased activity during lucid REM sleep contrasted with non-lucid REM sleep. Assignment of awareness criteria to brain regions: (1) Awareness of (spatial) orientation: 4, 5, 7, and 8; (2) Awareness of the capacity of choice: 1, 2, and 3; (3) Awareness of (intense) concentration – awareness of “flow”: 1, 2, and 3; (4) Awareness of identity (the “I”) : 4 and 6; (5) Awareness of the dreaming environment : 3, 4, 5, 7, and 8; (6) Awareness of the meaning of the dream : 1, 3, 4, and 5; Awareness of memory: 1, 3, 4, 5, and 6.

For each of the seven awareness criteria of lucid dreaming proposed, neurological evidence was collected. A visualization of our results can be seen in Figure 1. The most prominent feature of LD is the reactivation of brain areas that are inactive during regular REM-sleep dreams, which seem to explain the recovered awareness and consciousness of lucid dreamers. Awareness criteria nos. 1 and 2, the awareness of orientation and the awareness of being in charge, were considered essential for the experience of LD and accordingly, activation of relevant brain areas seems to exist. As Koch et al. (2016) suggested, multiple brain areas are involved in conscious experience, which include several frontal areas and a “posterior cortical hot zone.” The suggested emergence of a cortical network also points to brain plasticity and the fact that lucid dreaming can be learned and made easier by practicing. However, the findings presented above are not definite and should be further explored in the future. We do not want to imply that this attempt of explaining the underlying network of LD is the only or the best approach. Most studies used for reference have relied on small sample sizes, show low statistical power, discrepant results, and electrode montages in EEG studies were limited. Mota-Rolim et al. (2010) suggest that different subjective experiences and contents during lucid dreams might show different neurological activation. Changes in EEG might also depend on the LD experience of the dreamer and the vividness of a dream, individual working memory, emotionality, self-consciousness, as well as levels of attention and insight (Baird et al., 2019). As preliminary findings suggest, part of the observed activation of regions of *anterior prefrontal*, *parietal* and *temporal cortex* might not result from LD itself, but from the eye-signaling and hand-clenching task performed to signal lucidity, which also requires task-switching and sustained attention. Finally, we want to raise awareness for possible risks that might arise when practicing LD. While lucid dreaming can be a helpful tool in treating nightmares, depression or anxiety (Reynolds et al., 2006; Spoormaker and van den Bout, 2006; Doll et al., 2009; Holzinger et al., 2015) lucid dreams are also related to dissociative states, and phenomena like sleep paralysis, nightmares, or even psychosis or psychosis-like states might emerge in some cases (Holzinger, 2014; Aviram and Soffer-Dudek, 2018).

## Conclusion

Lucid dreaming has the ability to increase awareness and control of the dreamer. Neurological evidence seems to support the seven awareness criteria suggested by Holzinger. During LD, not a single brain structure, but a whole network of brain regions is activated. In this study, we hypothesize that the awareness criteria of LD proposed by Holzinger can be supported by empirical data. However, we want to make clear that we do not claim that this theory has already been proven, we merely use former findings to form our theory. Instead, we wish to push along further research based on Tholey’s theoretical concept. We think that theoretical and practical works regarding lucid dreaming make this approach very promising. Lucid dreaming shows potential as a methodology in the cognitive neuroscience of consciousness as well as psychotherapy (Zadra and Pihl, 1997; Holzinger, 2014; De Macedo et al., 2019). However, there is still substantial disagreement with regard to the brain regions and frequency bands most activated during lucid dreaming and how they correlate with the theoretical base of lucid dreams. Further research is needed.

## Author Contributions

BH and LM conducted the literature search, selected the eligible studies, and drafted the manuscript. Both authors confirm being the only contributors of this work and approved it for publication.

## Conflict of Interest

The authors declare that the research was conducted in the absence of any commercial or financial relationships that could be construed as a potential conflict of interest.
